# Pericardial Tamponade and Elevated Serum CA-125 Level in Inadequately Treated Hypothyroidism

**DOI:** 10.1155/2021/2666601

**Published:** 2021-11-10

**Authors:** Moaaz Baghal, Viralkumar Amrutiya, Bhoomi Patel, Rutwik Patel, Jonathan Hernandez, Mohamed Ahmed, Abraham Lo, Mark Gabelman

**Affiliations:** Hackensack Meridian Health Palisades Medical Center, Department of Internal Medicine, USA

## Abstract

Cardiac tamponade is a medical emergency and must be managed promptly, and reaching a diagnosis is imperative to prevent recurrence. Herein, we present a case of a young female patient that presented with progressive shortness of breath and abdominal distension and was found to have cardiac tamponade with the finding of elevation of a blood tumor marker, CA-125, in the setting of nonadherence to thyroid replacement therapy. She was managed by surgical pericardial window and abdominal paracentesis, with replacement of thyroid hormones leading to resolution of the tamponade and ascites. CA-125 elevation associated with cardiac tamponade and myxedema ascites due to hypothyroidism is very rare, and we aim to shed light on the importance of having a broad differential when approaching cardiac tamponade and understand the association between CA-125 and hypothyroidism.

## 1. Introduction

Primary hypothyroidism is prevalent in 4.6% of the US population, with a higher prevalence in women. According to the American Thyroid Association, reportedly one in eight women will develop a thyroid disorder [1]. The disease is usually life-long but can be well controlled with adherence to medical management. However, poor medical care or nonadherence can put the patient at risk for complications, which can range from mild to life-threatening, the latter of which can include the accumulation of fluids in the body cavities presenting as cardiac tamponade and myxedema ascites, which occur in 4% of mild cases, and can go up to 30-80% in severe cases [1, 2]. Cardiac tamponade secondary to hypothyroidism is rare as the fluid accumulates slowly, gradually progressing to pericardial sac distension [2]. Herein, we report a case of a young female patient with uncontrolled postthyroidectomy hypothyroidism in the setting of medication nonadherence who presented with elevated CA-125 levels, cardiac tamponade, and ascites, all of which were secondary to severe hypothyroidism.

### 1.1. Patient Information

A 31-year-old female with hypothyroidism secondary to total thyroidectomy for papillary thyroid carcinoma, with chronic medication nonadherence, presented with abdominal distension that had been progressing over the 3 months prior to admission. She also complained of worsening dyspnea which progressed to inability to finish her sentence, along with back pain radiating to her shoulder that started one hour prior to admission.

The patient had a history of papillary thyroid carcinoma status postthyroidectomy one year prior to admission, with the patient reporting a several-month history of medication nonadherence. The patient had no personal or family cardiac history.

### 1.2. Clinical Findings

Upon presentation, her blood pressure was 103/90 mmHg, heart rate ranged from 75 to 89 beats/min, respiration was 16 breaths/min, SpO_2_ was 98% on room air, and she was afebrile. Cardiovascular examination revealed a jugular venous pressure of 15 cm H_2_O, distant heart sounds, and no murmurs, and Kussmaul's sign was not present. However, pulsus paradoxus was not appreciated, and peripheral edema was absent. There was a well-healed horizontal neck scar from a previous total thyroidectomy, also known as a Kocher incision. She was noted to have mild facial swelling, mild generalized hypopigmentation over her skin, and enlarged nontender abdominal girth.

### 1.3. Diagnostic Assessment

**Figure 1:**
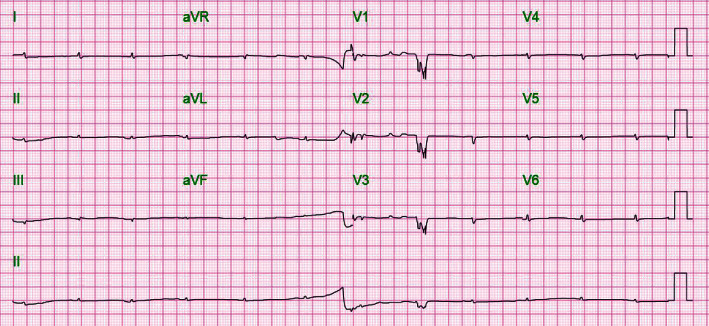
Electrocardiogram at presentation showing a sinus rhythm with a heart rate around 90 bpm and diffuse low voltage in both precordial and limb leads.

**Figure 2:**
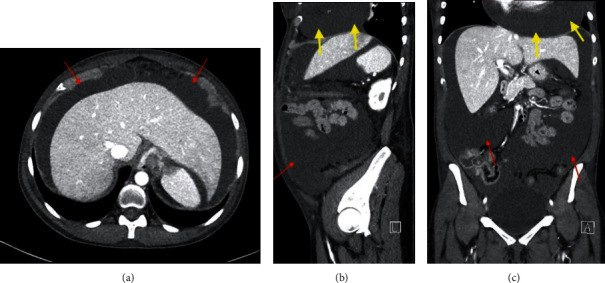
CT of the abdomen and pelvis with IV contrast on day 1. Mild bilateral basilar foci of atelectasis. Large pericardial effusion (yellow arrows in (b) and (c)). Large amounts of ascitic fluid (red arrows in (a)–(c)). Mild stranding of the peritoneum anteriorly.

**Figure 3:**
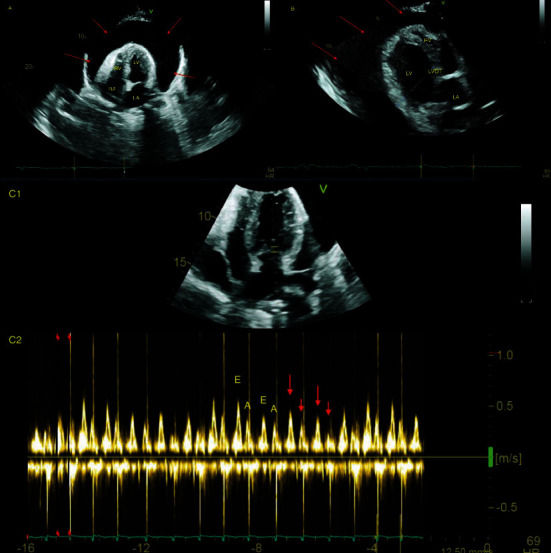
Transthoracic echocardiogram on day 0. Very large pericardial effusion with a swinging heart (red arrows in (a) and (b)), RV diastolic collapse, and marked mitral inflow respiratory variation (red arrows in (c)) consistent with significant tamponade. (a) Apical 4-chamber view; (b) parasternal long-axis view; (c) apical 4-chamber view showing the position of the Doppler pulse wave vector to obtain the mitral inflow waveform; (d) mitral inflow waveform.

Laboratory analysis revealed serum TSH level > 100 mIU/L and elevated levels of CA-125 of 230.1 U/mL. A diffuse low voltage electrocardiogram (EKG) (Figure 1) raised a suspicion for pericardial effusion, which was confirmed on initial enhanced computed tomography (CT) of the abdomen and pelvis, showing large pericardial effusion and peritoneal fluid collection (Figure 2). An urgent transthoracic echocardiogram (TTE) confirmed the very large pericardial effusion with a swinging heart, right ventricular collapse, and marked mitral inflow respiratory variation (Figure 3), for which the patient was taken for an emergent pericardial window with removal of 1.5 liters of serous fluid and pericardial drain placement. Pericardial fluid analysis and pericardial tissue demonstrated exudative effusion with reactive mesothelial cells, which were positive for calretinin and negative for tumor marker results such as MOC31, BerEp4, TTF1, and mucin. Pericardial effusion also had negative cultures and cytology.

It was initially difficult to reconcile such a profound presentation of fluid overload with a unifying diagnosis. Pregnancy was first ruled out. Along with hypothyroidism, a highly elevated CA-125 led to considering peritoneal and gynecologic malignancies at the top of the differential diagnosis. Autoimmune (e.g., SLE and rheumatoid arthritis); bacterial, fungal, and viral infections; and postviral complications were considered, along with congenital disorders (e.g., Wilson's disease).

### 1.4. Investigations

**Figure 4:**
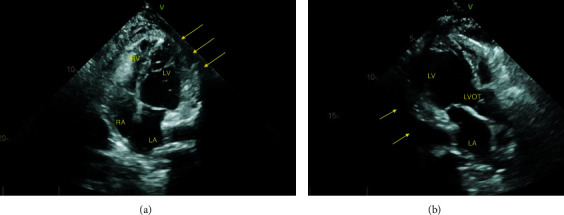
Transthoracic echocardiogram on day 1. The left ventricle has normal size. There is diffuse mild hypokinesis, LVEF is 45-50%. The right ventricular free wall appears thickened and echogenic. There is a small pericardial effusion. (a) Apical 4-chamber view and (b) parasternal long-axis view.

A follow-up TTE (Figure 4) demonstrated a small pericardial effusion, a thickened and echogenic right ventricular (RV) free wall, and diffuse mild hypokinesis of the left ventricle (LV) with an Ejection Fraction (EF) of 45-50%. Serum lactic acid was normal, and serum lipase was unremarkable. The iron profile showed low iron levels 47 *μ*g/dL with normal ferritin levels. B-Type Natriuretic Peptide (BNP) was 15 pg/mL. Initial serum troponin I levels were normal. Multiple serum tumor markers were tested; Carbohydrate Antigen (CA) 19-9 was 13 U/mL, CEA was 4.3 ng/mL, and Cancer Antigen (CA) 125 was 230.1 U/mL (reference: <35 U/mL). A SARS-CoV-2 rapid test was negative. Pericardial fluid analysis revealed a negative fungal culture, no mycobacterial species were isolated after 6 weeks of incubation, and bacterial culture was negative. Pericardial biopsy showed dense fibrosis and mesothelial cell changes, negative for malignancy. Peritoneal fluid analysis showed a SAAG of < 1.1/dL, and the rest of the peritoneal fluid studies were unremarkable. Serum lactate dehydrogenase was 162 U/L. Serum TSH levels were >100 *μ*IU/mL (reference: 0.34-5.6 *μ*IU/mL), serum free thyroxine level was <0.20 ng/dL (reference: 0.58-1.64 ng/dL), and total T3 level was <0.2 ng/mL (reference: 0.7-2.1 ng/mL). Serum thyroglobulin level was less than 0.4 ng/mL. Serum anti-thyroglobulin antibody level was 6 IU/mL (reference < 1). Pelvic vaginal ultrasound did not reveal any signs of ovarian mass or uterine pathology. The serum rheumatoid factor was negative. Serum ANA was negative. Serum ceruloplasmin and serum copper levels were normal. Epstein-Barr virus IgM and IgG were negative. Coombs' test was negative.

### 1.5. Therapeutic Intervention

Postprocedure, the size of the pericardial effusion remained stable. The condition was complicated by left-sided pneumothorax, which was considered a postprocedure complication, and required chest tube placement and admission to the critical care unit, where she was monitored. For hypothyroidism, the patient was started on thyroid replacement therapy. On day 1, a paracentesis was performed with removal of 3 liters of fluid from the abdomen. The patient was closely monitored, and she remained hemodynamically stable on day 2; the pneumothorax was resolved with subsequent removal of the chest tube, and the patient was downgraded to medical floor for further medical care. The patient was later discharged home on levothyroxine with strict recommendations on the importance of adherence and outpatient follow-up.

### 1.6. Follow-Up and Outcomes

**Figure 5:**
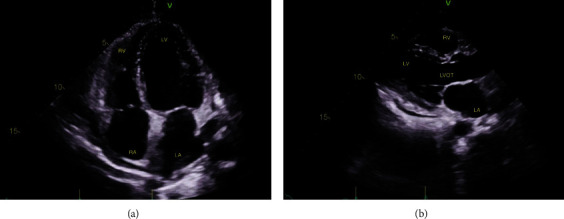
Transthoracic echocardiogram 4 weeks since admission. The left ventricle has normal size. LVEF is 55-60%. LV strain is -19%. The right ventricle free wall is thick, and mitral valve leaflets are thickened. Small-to-moderate circumferential pericardial effusion. There is no evidence of tamponade. (a) Apical 4-chamber view and (b) parasternal long-axis view.

A close follow-up of our patient in the outpatient setting showed normal levels of TSH with no signs of recurrent malignancy on follow-up ultrasound of the neck. Follow-up transthoracic echocardiogram 4 weeks later showed improved LV and RV systolic function with improved LVEF with mild pericardial effusion and no evidence of pericardial effusion (Figure 5). Her CA-125 levels were trending down to 124.8 U/mL, and the patient reported no further symptoms on adherence to thyroid replacement therapy. An outpatient PET scan with the oncology workup returned negative (Table 1).

## 2. Discussion

Depending on the severity of hypothyroidism, cardiac function can be impacted caused by changes in the cardiovascular system due to low T3 levels, leading to decreased cardiac output, increased diastolic blood pressure, narrowing of the pulse pressure, and bradycardia; in severe hypothyroidism, it can lead to pericardial effusion [2].

The mechanism of hypothyroidism-induced pericardial effusion is unclear, but it is believed to be associated with increased capillary permeability and reduced lymphatic drainage from the pericardial space [2]. It is hypothesized that the low synthesis of albumin, which usually occurs 2 to 3 months after a hypothyroid state, with increased interstitial pressure plays a major role [3]. In addition, it is believed that the increased extravascular accumulation of protein is a result of increased outward transcapillary transport rate of albumin and poor compensatory increased lymphatic flow with slow protein return rate to the intravascular space. Only in 4 percent of cases, extravascular plasma protein accumulation occurred in the heart, liver, kidneys, and spleen [4]. Pericardial effusion and cardiac tamponade are acutely managed by pericardiocentesis; in some cases, a prolonged pericardial drainage and a pericardial window are reverted in case of reaccumulation and signs of loculation, or biopsy material is required, as per the latest ESC 2015 guidelines. In our patient's case, the diagnosis of pericardial effusion in the setting of malignancy was suspected, and it was the cardiothoracic surgeon's decision to perform a pericardial window with pericardial drainage and collect the pericardial fluid and sac for pathological and cytological testing [3].

Noncardiac manifestations, such as ascites as in our patient's case, have had the same pathophysiological hypothesis as pericardial effusion. The ascitic fluid analysis showed a SAAG of 1.0 g/dL, indicating that the cause is not due to portal hypertension. As ascites is not considered a common complication of hypothyroidism, the suspicion for hypothyroidism was low and other causes needed to be excluded. Our patient's condition coupled with elevated levels of CA-125 warranted a workup for ovarian cancer as hypothyroidism has also been rarely associated with elevated CA-125 levels, with one case reported in Japan in 2007 [5].

**Figure 6:**
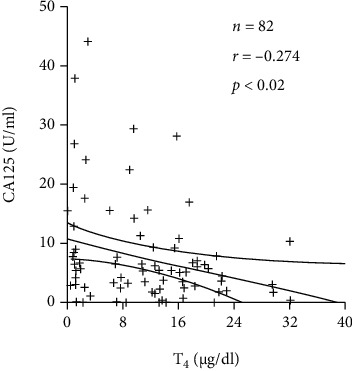
Graph showing the correlation between CA-125 and T4 concentrations in patients with altered thyroid states, demonstrated by Hashimoto et al. in their published article. Changes in tumor marker concentration in female patients with hyper-, eu-, and hypothyroidism [9].

CA-125 was first described in 1981 by Bast et al. as a glycoprotein raised in 90% of advanced ovarian tumors and has since been used prospectively as a marker for therapeutic efficacy and disease monitoring among ovarian cancer patients but has not been used for screening for ovarian cancer due to inadequate sensitivities, as proven by previous studies [6]. It is rare for hypothyroidism to be correlated with elevated levels of CA-125 [4], with the exact mechanism not being identified yet. One hypothesis explains that the elevation of CA-125 occurs in cases of stretching of the peritoneum with ascitic fluid accumulation, and elevated CA-125 can reach levels comparable to those in ovarian carcinoma; such correlations have been seen in nephritic syndrome and liver cirrhosis, as described by Castro et al. [7]. Another hypothesis is the elevation of CA-125 due to pericardial effusion with delayed clearance of CA-125 in hypothyroid states, with the possibility of CA-125 being related to the severity of hypothyroidism-associated pericardial effusion. A study done by Hashimoto et al. in 1989 showed an inverse relationship between the levels of T4 concentration in abnormal thyroid states and CA-125 (Figure 6) [8, 9].

Typical features of myxedema ascites include long duration of hypothyroidism and rapid resolution of ascites following thyroid hormone replacement therapy. In cases of ascites, the diagnostic workup includes paracentesis to analyze the ascitic fluid and to narrow the differential diagnosis, which can be divided into high protein ascites, which usually includes peritoneal malignancies, tuberculous peritonitis, pyogenic peritonitis, and pancreatic ascites, and low protein ascites, which is correlated with liver cirrhosis and congestive heart failure [10].

Peritoneal malignancy must be suspected when the composition of ascitic fluid and ultrasonography are not consistent with portal hypertension. Although in our case, we detected an elevated CA-125, workup for peritoneal malignancy and ovarian carcinoma was done while restarting thyroid hormone replacement therapy, performing a pericardial window for cardiac tamponade and paracentesis for ascites with a positive response noted in the patient's condition. When the initial workup for malignancy came back negative, the definitive answer would come in the outpatient follow-up setting, where close follow-up and patient adherence to her thyroid medication led to complete resolution of her presentation with no recurrence, and no evidence of peritoneal malignancy was found as well as excluding all other causes.

Early diagnosis of the causes of ascites and cardiac tamponade would prevent the inappropriate use of other management modalities such as frequent paracentesis, liver biopsies, and in cases of failure of diagnosis an exploratory laparotomy [5]. However, not all cases of hypothyroidism-associated ascites present with a SAAG < 1.1, as Oey et al. [4] presented in their study review that the patient can also present with a SAAG > 1.1. Therefore, levels of SAAG are variable, and neither high nor low SAAG has shown to be a constant feature of myxedema ascites.

Adequate history, physical examination, and guideline-directed management are essential for the early diagnosis of cardiac tamponade and myxedema ascites. Although the condition is rare, it is easily reversible and avoidable with thyroid hormone replacement therapy.

We report our experience to shed light on the importance to exercise caution when analyzing elevated tumor markers in patients with abnormal thyroid hormone levels and to consider the thyroid to be correlated with elevated CA-125 and fluid overload when other devastating causes have been ruled out without having to perform unnecessary invasive procedures. More studies are still needed to understand the mechanisms of pericardial effusion and elevated CA-125 in hypothyroidism.

## Figures and Tables

**Table 1:** Timeline.

Day 0	Patient undergoes surgical pericardial window, complicated postoperatively by left-sided pneumothorax that required chest tube placement. Thyroid replacement therapy started
Day 1	Paracentesis performed, with removal of 3 liters of fluid
Day 2	Resolution of pneumothorax with removal of the chest tube
Day 5	Discharged home on strict instructions to adhere to thyroid medication and follow-up with cardiology, obstetrics and gynecology, and endocrinology
Day 37	Outpatient follow-up showed downtrending of CA-125 levels. Follow-up echocardiogram revealed no recurrence of pericardial effusion. Thyroid hormone levels were within the normal range

## Additional Points

*Patient Perspective*. The patient was contacted and declined to share her perspective on the case.
